# Supplementary material for: A longitudinal assessment of the antibody response to SARS-CoV-2 infection in the New Mexican population
Source: PLoS One. 2025 Jul 8;20(7):e0327698. doi: 10.1371/journal.pone.0327698 (PMC12237266; doi:10.1371/journal.pone.0327698)
Supplement: Attachment — Submitted filename: Response to Reviewers.docx [file pone.0327698.s002.docx]

**Response to Reviewers**

We did not receive any feedback from any reviewers, suggesting that none were obtained during the 3.5 month interval that PLOS ONE was in receipt of our manuscript. The only comments we received from the academic editor was:

“After careful consideration, we have decided that your manuscript does not meet our criteria for publication and must therefore be rejected”

and

“The article was well written but it is preferable to publish in your regional country.”

Our responses:

1) There are no criteria in the PLOS ONE “Criteria for Publication” that includes reasoning that some studies are better served being published in a “regional country.” This reasoning appears to be a clear violation of PLOS ONE policy.

2) Our study occurred in New Mexico, which has been a state in the United States of America since 1912. (Our article clearly states that New Mexico is part of the US in the introduction; we have added this as well to the absract). It was the 47^th^ state, and was admitted before Arizona, Alaska, and Hawaii. The idea that we should be publishing in our “regional country” would mean we should be publishing in PLOS ONE, which is based in the US. However, in case there is a requirement for proving that a study in New Mexico deserves to be published in the US, here are some fun facts about New Mexico:

a) New Mexico is the 5^th^ largest state by size in the US.

b) New Mexico, despite being 37^th^ in population, has the most human hantavirus cases in the US.

c) New Mexico is home to 2 US national labs (Sandia National Laboratories and Los Alamos National Laboratory).

d) New Mexico is home to 3 US air force bases (Cannon, Holloman, and Kirtland). Many of the original stealth fighter aircraft were housed at Holloman.

e) New Mexico is the 2^nd^ largest oil-producing state in the US and is 7^th^ in the US in wind power.

f) The first nuclear bomb was developed and tested in New Mexico.

g) New Mexico is a major filming site in the US, known for such shows as “Breaking Bad,” “Better Call Saul,” and “Oppenheimer.”

h) The capital of New Mexico, Santa Fe, is the oldest state capital in the US, founded in 1610.

i) New Mexico is one of 7 states that have a majority-minority population.

j) New Mexico has the 3^rd^-highest percentage of Native American citizens of any state in the US.

k) New Mexico produces 33% of chile and 29% of pecans in the US.

l) New Mexico hosts an annual hot air balloon fiesta that is the largest in the world.

Therefore, we feel that our manuscript should be reviewed and is suitable for publication in PLOS One.
