# Supplementary material for: A longitudinal assessment of the antibody response to SARS-CoV-2 infection in the New Mexican population
Source: PLoS One. 2025 Jul 8;20(7):e0327698. doi: 10.1371/journal.pone.0327698 (PMC12237266; doi:10.1371/journal.pone.0327698)
Supplement: Attachment — Submitted filename: Response_June3_2025_SB.docx [file pone.0327698.s003.docx]

Response to Reviewers

We would like to thank the reviewers for the thoughtful responses to our manuscript. We have made the changes outlined below to address the reviewers’ and journal’s comments. Our responses are below in *italics.*

Reviewer 1:

The introduction section inappropriately includes extensive information regarding the study methodology and main results. Specifically:

The recruitment timeline, patient demographics, and cohort description (lines 36-53).

Key results related to IgG, NAb, and ADCC findings (lines 91-110) are presented here.

Information about the circulating SARS-CoV-2 strain (Wuhan) and the study design (two blood draws) should be moved to the Materials and Methods section.

The introduction should be refocused on providing background, identifying the knowledge gap, and stating the study objective without introducing data or methods.

*We have moved the recruitment timeline, patient demographics, and cohort description from the Introduction to the beginning of the results section (lines 193-204). Information regarding the recruitment timeline and cohort description are also included in the Methods section.*

*We have removed key results relating to IgG, NAb, and ADCC findings from the Introduction. These findings are outlined in the Results section as suggested by reviewer 1.*

*Information regarding the circulating strain (Wuhan) has been moved to a subsection in Materials and Methods entitled “Viruses and Antigens” (lines 113-117).*

*Information about the study design have been removed from the Introduction. This information can be found in the “Human Subjects” subsection of the Materials and Methods section.*

*We have refocused the Introduction section on providing background and emphasizing the study objective.*

Incomplete reporting of key experimental details:

The authors mention using HEK293 cells overexpressing the Wuhan-Hu-1 S gene for ADCC assays but do not report the validation of the tecnic for the expression of the S protein (e.g., by flow cytometry or Western blot). Given the reliance on these target cells, internal validation should be either performed or referenced.

*The expression of the S protein was identified by the manufacturers through flow cytometry. We have included an explanation of this expression validation in the “Cell Lines” subsection of Materials and Methods. We have also included a photo of this validation below:*

Lack of information on statistical correction:

There is no mention of adjustment for multiple comparisons (e.g., Bonferroni or FDR) despite multiple statistical tests (IgG, NAb, ADCC across groups and timepoints).

*We have included information on the adjustment for multiple comparisons (Dunn’s tests) as well as information on the software used for statistical analyses in the “Statistical Analysis” subsection of the Materials and Methods section (lines 187-189).*

While technically sound, the results section would benefit from a subgroup analysis based on vaccine type (Pfizer-BioNTech, Moderna, Janssen), as this could influence immune responses. This omission limits the interpretability of vaccination-related findings.

There is no mention of comorbidities in the population of the study.

*We have added vaccine information in the Results section (lines 211-213). We have also included information about co-morbidities in our study (lines 214-215). Vaccine type, ethnicity, and co-morbidities were self-reported. We have added a description of this in the “Human Subjects” subsection of the Materials and Methods section (line 79).*

The sample size in some subgroups (e.g., unvaccinated N=11, vaccinated N=14) is modest, which restricts the statistical power for detecting differences, particularly in ADCC data where variability is expected.

We have added text to address this concern in lines 443-446 of the Discussion section.

In the results, you grouped participants simply as "vaccinated" without distinguishing whether there were differences among those who received different types of vaccines.

The manuscript would be stronger if you mentioned that you attempted to compare vaccine types, or if you acknowledged that the sample size limited the ability to perform sub-analyses by vaccine type. This is important because each vaccine elicits distinct humoral and Fc-mediated immune responses.

*We have included language about how our sample size limited our ability to perform sub-analyses by vaccine type (as well as co-morbidity) in the Results (lines 257-258) and the Discussion (lines 446-448).*

The discussion does not fully address some key limitations:

There is no discussion on the lack of vaccine-type stratification in the vaccinated group.

The authors acknowledge the limited cohort size in the ethnic comparison but should also emphasize how this impacts statistical power across all comparisons (e.g., antibody functional assays).

*We have addressed this in lines 443-446 of the Discussion section.*

The discussion would also benefit from a clearer statement regarding the scope and applicability of the findings. For example, the authors should elaborate on how their results could be generalized (or not) to other populations or settings beyond the New Mexican cohort, and the potential relevance of these findings to current SARS-CoV-2 variants or vaccination strategies.

*We have included language about the scope of this study in lines 450-456 of the Discussion section.*

The discussion could also benefit from reflecting on the potential implications of using reporter cell lines (Jurkat-Lucia™ NFAT-CD16) versus primary NK cells in ADCC assays, which is relevant for interpreting effector function results.

*We have described the implications of using our reporter cell line versus primary NK cells in ADCC assays in lines 425-434 of the Discussion section.*

Reviewer 2:

Perhaps it would be helpful if the authors, if they have the information, could comment on whether their cohort used only one type of vaccine, or several, and if so, which ones were available to them at that time.

*We have included this information in the Results section (lines 211-213).*

Table 2 includes an unusual column interpreting p values as non-significant. Perhaps the column with the p value could be left alone and left to each reader to interpret it.

*We have removed the column interpreting p-values as non-significant (lines 349-355)*

Some journals require that the p value have only a few values after the decimal point; it is worth review it.

*We have reviewed PLOS ONE’s policies on reporting of p-values and have not observed any requirements about values after the decimal point.*

Journal:

Please ensure that your manuscript meets PLOS ONE's style requirements, including those for file naming. The PLOS ONE style templates can be found at

https://journals.plos.org/plosone/s/file?id=wjVg/PLOSOne_formatting_sample_main_body.pdf and

https://journals.plos.org/plosone/s/file?id=ba62/PLOSOne_formatting_sample_title_authors_affiliations.pdf

*We have ensured that our manuscript meets PLOS ONE’s style requirements, including those for file naming.*

Thank you for stating the following financial disclosure:
“UNM intramural grant CTSC003-11”
Please state what role the funders took in the study. If the funders had no role, please state: "The funders had no role in study design, data collection and analysis, decision to publish, or preparation of the manuscript." If this statement is not correct you must amend it as needed.
Please include this amended Role of Funder statement in your cover letter; we will change the online submission form on your behalf.

*The funders had no role in study design, data collection and analysis, decision to publish, or preparation of the manuscript. Materials were obtained through the 203298 Covid 19 Research Fund.*

Please note that funding information should not appear in the Acknowledgments section or other areas of your manuscript. We will only publish funding information present in the Funding Statement section of the online submission form. Please remove any funding-related text from the manuscript.

*Funding information has been removed from the manuscript.*

We note that your Data Availability Statement is currently as follows:
“All relevant data are within the manuscript and its Supporting Information files”
Please confirm at this time whether or not your submission contains all raw data required to replicate the results of your study. Authors must share the “minimal data set” for their submission. PLOS defines the minimal data set to consist of the data required to replicate all study findings reported in the article, as well as related metadata and methods (https://journals.plos.org/plosone/s/data-availability#loc-minimal-data-set-definition).
For example, authors should submit the following data:
- The values behind the means, standard deviations and other measures reported;
- The values used to build graphs;
- The points extracted from images for analysis.
Authors do not need to submit their entire data set if only a portion of the data was used in the reported study.
If your submission does not contain these data, please either upload them as Supporting Information files or deposit them to a stable, public repository and provide us with the relevant URLs, DOIs, or accession numbers. For a list of recommended repositories, please see https://journals.plos.org/plosone/s/recommended-repositories.
If there are ethical or legal restrictions on sharing a de-identified data set, please explain them in detail (e.g., data contain potentially sensitive information, data are owned by a third-party organization, etc.) and who has imposed them (e.g., an ethics committee). Please also provide contact information for a data access committee, ethics committee, or other institutional body to which data requests may be sent. If data are owned by a third party, please indicate how others may request data access.

*We have submitted our data set to DRYAD:*

http://datadryad.org/share/1DxzK9y9c_IdS4QU9F2asirTrmQEQ-pzDwtLqSP863s
